# Nanoparticle mediated targeting of VEGFR and cancer stem cells for cancer therapy

**DOI:** 10.1186/2045-824X-3-26

**Published:** 2011-11-14

**Authors:** Rashmi K Ambasta, Archita Sharma, Pravir Kumar

**Affiliations:** 1 Functional Genomics and Cancer biology lab, CME, VIT University, Vellore, India; 2 Adjunct Faculty, Tufts University School of Medicine, Boston, MA, USA

**Keywords:** Nanoparticle, Cancer stem cell, VEGF, Cancer therapy

## Abstract

Angiogenesis is a crucial process in tumor pathogenesis as it sustains malignant cells with nutrients and oxygen. It is well known that tumor cells secrete various growth factors, including VEGF, which triggers endothelial cells to form new capillaries. Prevention of expansion of new blood vessel networks results in reduced tumor size and metastasis. Production of VEGF is driven by hypoxia via transcriptional activation of the VEGF gene by HIF-1α.

Tumours are now understood to contain different types of cells, and it is the cancer stem cells that retain the ability to drive the tumour's growth. They are called cancer stem cells because, like stem cells present in normal tissues of the body, they can self-renew and differentiate. These cancer stem cells are responsible for the relapse of cancer as they are found to be resistant to conventional modes of cancer therapy like chemotherapy and radiation.

In this review, a novel mode of treatment of cancer is proposed, which utilizes the twin nanoparticle to target endothelial cells in the niche of cancer stem cell. The nanoparticle discussed in this review, is a twin nanoparticle of iron coated with gold, which targets VEGF positive cell in the vicinity of cancer stem cell. In the twin nanoparticle, one particle will recognize cancer stem cell, and another conjugated nanoparticle will recognize VEGF positive cells, thereby inhibiting endothelial cells in the proximity of cancer stem cell. This novel strategy will inhibit angiogenesis near cancer stem cell hence new tumour cannot grow and old tumour will be unable to metastasize.

## Introduction

Angiogenesis is the formation of new blood vessels from the pre-existing vasculature. It is an important process as the blood delivers oxygen and nutrients for the survival of cells and their functioning. It has been reported that in the absence of blood vessel formation, the tumor mass does not grow more than 1-2 mm in size and also the cells from the primary tumor cannot escape and metastasize.

Angiogenesis is controlled by pro-angiogenic and anti-angiogenic factors in the body. The regulatory factors may be several growth factors like VEGF, FGF, PDGF, EGF, placental growth factor, Angiopoietin-1, Angiogenin, Interleukin 8 etc. The natural anti-angiogenic factor presents in the body are Angiostatin, Endostatin, Vasostatin, Prolactin, Angiopoietin-2, Interferon, -α and γ Interleukin 12, Fibronectin, Platelet factor-4, etc. A finely tuned equilibrium exists between these pro-angiogenic and anti-angiogenic factors *in vivo *[1].

**Figure 1:**
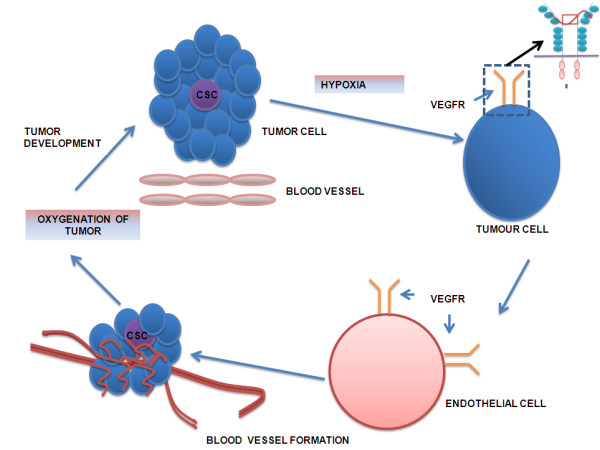
**A pathway regulating tumour growth i.e. cancer stem cells trigger tumour growth in response to hypoxic condition and releases the signal VEGF**. The VEGF initiates the endothelial cell to proliferate and form blood vessels to vascularize the growing tumour. As soon as the tumour gets oxygenated, the tumour grows further in size.

Angiogenesis is driven due to hypoxic conditions in the tumor. The hypoxic condition activates the production of VEGF (Vascular Endothelial Growth Factor) which then binds to the VEGF receptor (VEGFR), a transmembrane receptor tyrosine kinase on the membrane of endothelial cells and activates them by phosphorylation of tyrosine kinase (TK). The TKs are also present on the receptors of FGF, PDGF, Ang-1, Ang-2, and HGF along with that of VEGF and leads to angiogenesis [1]. The activation of VEGFR on endothelial cells leads to the formation of a blood vessel and oxygenation of tumour, which in turn leads to growth of tumour size (as shown in Figure 1). We are especially focusing on VEGF as it has been well proven to inhibit tumour growth upon tagging with single nanoparticle. The role of other growth factors has not been well studied so far to prove that they alone can inhibit tumour growth. In this review, we are discussing about the role of VEGF in tumor progression near cancer stem cells and targeting it using twin nanoparticles.

## VEGF Inhibitors

VEGF is a heparin binding homodimeric glycoprotein that binds to endothelial specific transmembrane receptors tyrosine kinases. The VEGF family contains different types of VEGF like, VEGF-A, VEGF-B, VEGF-C, VEGF-D, VEGF-E and P1GF (placenta growth factor). The receptors of VEGF are named as Flt-1 (VEGFR-1), KDR/Flk-1 (VEGFR-2), FLT-4 (VEGFR-3) [2]. VEGFR-1 and VEGFR-2 are involved in angiogenesis whereas the VEGFR-3 is involved in lymphangiogenesis [3].

A number of studies have shown that the overexpression of VEGF and VEGFR are associated with tumor growth, progression and metastatis. Metastatic tumor has been known to secrete VEGF to induce the neovascularization [4]. There are three types of VEGF receptors namely VEGFR-1, 2 and 3 and there are different types of ligand that binds to these three receptors as listed in Table 1. VEGF A, B and P1GF binds to VEGFR-1 [5-8]. VEGF A, B,C,D binds to VEGFR-2 [9-11] and VEGF C and D binds to VEGFR-3 [12,13].

**Table 1:** Types of VEGFR and VEGF

Type of VEGFR	Binds to	Location of expression	Reference
VEGFR-1	VEGF-A, VEGF-B, P1GF	Thymus, monocytes, macrophages, dendritic cells, osteoclast, astrocytes	[5-8]
VEGFR-2	VEGF-A, VEGF-B, VEGF-C, VEGF-D	Vascular endothelial progenitors cells, heamatopioetic stem cells, Neuronal cells, osteoblasts, osteoclast, pancreatic duct cells, retinal progenitor cells, megakaryocytes	[9-11]
VEGFR-3	VEGF-C, VEGF-D	Lymphatic endothelial cells, capillaries and veins in endocrine organs, monocytes and macrophages, neural progenitor cells	[12,13]

We understand that different tumour express different types of VEGF and VEGFR. Table 2 also shows that different VEGF inhibitors have been targeted against different VEGFR till now. Therefore, twin nanoparticle will target only the tumour specific VEGF and VEGFR.

**Table 2:** List of VEGF Inhibitors

Agent	Class	Target	References
Bevacuzimab	MAB	VEGF-A	[17,18]
IMC-1121B	MAB	VEGFR-2	[19]
CDP-791	DiFabPEG	VEGFR-2	[20]
CEP-7055	TKI	VEGFR-1,2,3	[20]
PTK-787	TKI	VEGFR-1,2	[21]
AEE788	TKI	VEGFR-2, EGFR	[22]
ZD6474	TKI	VEGFR-1,2,3, EGFR	[23]
AG013736	TKI	VEGFR-1,2	[24]
AZD2171	TKI	VEGFR-1,2	[25]
SUO11248	TKI	VEGFR-1,2, PDGFR	[26]
CP-547,632	TKI	VEGFR-1,2	[27]
GW786024	TKI	VEGFR-1,2,3	[28]
Bay 93-4006	TKI	VEGFR-1,2, PDGFR	[29]
AMG 706	TKI	VEGFR-1,2,3	[30]

The VEGF pathway is a good target for the anti-angiogenic therapy for various reasons like-

1. It is produced in large quantities by growing primary tumors.

2. VEGF pathway induces the production of sprouting blood vessels.

3. VEGF binds to endothelial cells involved in the formation of blood vessels. Also endothelial cells are genetically stable and spontaneous mutations are rare when compared to unstable tumor cells.

4. VEGFR are expressed in low levels in normal cells, and extensively in tumor cells [14].

Several preclinical studies and clinical trials of phase I and phase II using both monoclonal antibodies against VEGF and VEGFR pathways have proven that these agents are safe. These agents are also of great potential in cancer therapy in patients by blocking the angiogenesis pathway thus restricting the tumor mass and reducing the possibility of metastasis [15,16].

There are various inhibitors available which target the VEGF pathway. Bevacuzimab is a humanized monoclonal antibody which binds to VEGF-A [17,18] before it can attach to its receptor VEGFR1 and VEGFR2, thus blocking the pathway. Another monoclonal antibody used to target VEGF pathway is IMC-1121B which selectively binds and inhibits the VEGFR-2 [19]. It is a chimeric IgG1 antibody and its binding prevents the formation of ligand- receptor complex and thus inhibits the dimerization and phosphorylation of tyrosine kinase. There are several tyrosine kinase inhibitors which prevent the phosphorylation of the tyrosine kinase thus blocking the angiogenesis. Some of the examples are given in Table 2 like, CDP-791 targets VEGFR-2 [20] and there are several tyrosine kinase inhibitors namely PTK-787 [21], AEE788 [22], ZD6474, AG013736 [23,24], AZD2171 [25], SUO11248 [26], CP-547,632 [27], GW786024 [28], Bay 93-4006 [29], AMG 706 [30].

Bevacuzimab has shown good tolerance in patients in preclinical and early clinical studies. Although it has shown some side effects such as hypertension and risk of thromboembolism due to toxicity, but clinical efficiency has also been demonstrated [31].

The VEGF inhibitors will not be specific to the tumor cells as the normal cells also contain VEGF and VEGFR though in less concentration than in the tumor cells where it is overexpressed due to hypoxic conditions. These hypoxic conditions lead to the expression of a transcription activator hypoxia inducible factor alpha (HIF-1α). HIF-1α has also been found in cancer stem cells and hence it is an effective way of targeting the drug or monoclonal antibody via the use of nanoparticle [32]. The nanoparticle could be gold, iron, which encapsulates the drug/monoclonal antibody [33]. Although VEGF/VEGFR is also found in normal cell, one cell i.e. unique to tumour is cancer stem cell. Therefore, it is important to target VEGF in the vicinity of cancer stem cell(s).

## Cancer stem cells and their markers

The most exciting research in the cancer therapy is the targeting of cancer stem cells (CSC). There has been evidence that the growth and propagation of the cancer is dependent on the small subset of cells which are cancer stem cells [34]. These stem cells are present in the stem cell niche which functions to maintain these cells. Several extrinsic factors are generated by these niches which are responsible for stem cell growth and proliferation and play an important role in a self-renewal and regulate the fate of lineage [35,36]. Stem cells also have the higher degree of proliferation, a longer life span compared to their progeny and a higher tendency to undergo mutation. Any mutation in the pathways that control these stem cells may lead to impaired stem cell function and thus leading to uncontrolled proliferation and impaired differentiation. These cells are also believed to be involved in the relapse of the cancer in an individual for e.g. in hematopoietic malignancies. These stem cells are similar to normal stem cells in the following ways- 1) they have the ability to self- renew, 2) they have the potential to differentiate into heterogeneous daughter cells, 3) they can proliferate extensively and give rise to a complete new tumour from one cell [37]. These CSCs are highly tumourigenic and the main concern about them is that they are resistant to conventional treatment like chemotherapy [38,39]. This has been stated as the cause for tumor relapse as these cells are not destroyed [40]. VEGF is also targeted in the niche vasculature with the help of drugs like Bevacizumab [41], but the major problem is a blockage of VEGF or angiogenesis in normal cells also [42,43]. Therefore, in this review, we try to design a novel cancer therapy method that can be used to target angiogenesis near these cancer stem cells to prevent the recurrence of tumor. Although, there is a problem in recognizing tissue-specific cancer stem cell, we have a list of cancer stem cell marker available based on the research conducted, until now. These markers may be a novel target for targeting the CSCs and avoiding the possibility of recurrence and metastasis in various carcinomas.

There are different types of cancer stem cell marker which varies with the organ. Some lists of the organ specific cancer stem cells markers which are present on the surface are summarized in Table 3. CD44/CD24, CD29, CD133, CD200 are the cancer stem cell marker for breast cancer [44-47]. CD44, CD24, ESA, CD133, CXCR4 are the cancer stem cell marker for pancreatic cancer [48-50]. Stro-1, CD105, CD44 are the cancer stem cell marker for bone cancer [51]. EpCAM, CD44, CD166, CD133 are the cancer stem cell marker for colorectal cancer [52,53]. CD133, CD44, Integrin 12b1 are the cancer stem cell marker for prostate cancer [54]. CD90, CD133 are the cancer stem cell marker for liver cancer [55]. CD133, CD200 are the cancer stem cell marker for brain cancer [56,57]. CD44, BMI-1 are the cancer stem cell marker for head and neck cancer [58,59].

**Table 3:** List of cancer stem cell surface markers

Type of Cancer	Cancer Stem Cell Marker	References
Breast Cancer	CD44/CD24, CD29, CD133, CD200	[44-47]
Pancreatic Cancer	CD44, CD24, ESA, CD133, CXCR4	[48-50]
Bone Cancer	Stro-1, CD105, CD44	[51]
Colorectal Cancer	EpCAM, CD44, CD166, CD133	[52,53]
Prostate Cancer	CD133, CD44, Integrin 12b1	[54]
Liver Cancer	CD90, CD133	[55]
Brain tumour	CD133, CD200	[56,57]
Head and Neck	CD44, BMI-1	[58,59]

**Figure 2:**
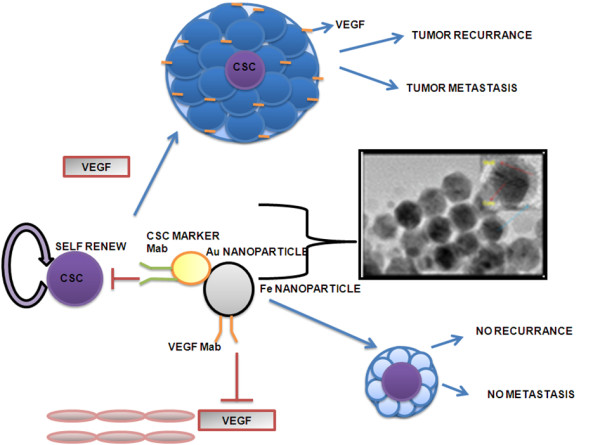
**The above diagram shows that usually the cancer stem cells gives rise to new tumour in the presence of VEGF or neovascularization in the proximity of cancer stem cells**. The novel therapy procedure that targets the endothelial cell in the proximity of cancer stem cell will lead to neither tumour growth nor metastasis.

In this review we are discussing about a nanoparticle which is specific to cancer stem cells markers and also carries VEGF monoclonal antibody, thus inhibiting the process of angiogenesis in the vicinity of cancer stem cells. This strategy will reduce the recurrence of cancer as it inhibits angiogenesis in proximity of cancer stem cells as shown in Figure 2, thereby inhibiting the new tumour formation.

### Nanoparticle mediated therapy

Nanotechnology is the study of devices that are in the dimensional range of 1-1000 nm. Nanotechnology has been frequently used in oncology for the delivery of drugs. The various methods of drug delivery in cancer therapy using nanotechnology are nanoparticles, liposomes, polymeric micelles, dendrimers, nanocantilevers, carbon nanotubes and quantum dots. The concept of using these nanoparticles for the purpose of drug delivery was first proposed by Widder, Senyi and colleagues in 1978. The basic principle is that the drug is either attached on or encapsulated within the nanoparticle. These particles may have a polymer or metal coating with magnetic cores. It is possible to attach or immobilize the drug or an antibody on its surface and target it to the desired site. If the nanoparticle is magnetic in nature then it can be manipulated by applying a magnetic field from an external source. Magnetic drug targeting is an emerging field in the cancer therapy. Gold shell and iron core nanoparticle have been synthesized to carry drugs (for example; Doxorubicin which binds to amino group in the gold shell). Another method of drug delivery has recently been developed by the scientists in Brown University by coupling of magnetic nanoparticle and gold nanoparticle with specific markers and creating a twin nanoparticle [60-62].

They created a twin nanoparticle which specifically binds to Her-2 receptors and then unloads the drug in the breast cancer cells. This has been successfully demonstrated in cancer cell lines. The nanoparticle can be concentrated in the tumor region by magnetic property and the specific drug or monoclonal antibody can be targeted and released specifically at the tumor site without affecting any other part of the body. This shall result in minimum level of side effects and toxicity in the body. Also the magnetite and gold are both stable and non toxic. In this review we propose to design a twin nanoparticle i.e. iron core gold shell nanoparticle [63-66].

### Design of SPIONS@Au nanoparticle

Gold nanoshell around super paramagnetic iron oxide nanoparticles (SPIONs) can be synthesized with a gold coating of approximately 0.4 to 0.5 nm thickness. The gold-coated SPIONs (SPIONs@Au). Recently, it has been demonstrated that in the absence of oscillating magnetic field, both SPIONs and SPIONs@Au are not particularly cytotoxic to mammalian cells in culture [67,68]. However, nobody has tried these nanoparticles for cancer therapy. We propose to tag the iron with VEGF monoclonal antibody and gold with cancer stem cell marker.

**Figure 3:**
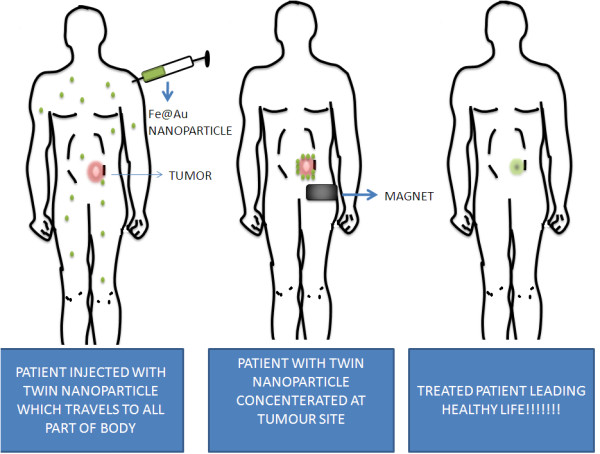
**The above figure demonstrates a twin nanoparticle made of iron oxide and gold which are coupled together along with cancer stem cell marker and VEGF monoclonal antibody**. The nanoparticle concentrates on the tumour site using magnet and cancer stem cell marker and then releases VEGF monoclonal antibody so that angiogenesis can be inhibited near cancer stem cell and tumour growth can be stopped.

The twin nanoparticle can be mobilized to the tumour region by applying external magnetic field so that it does not affect the whole body as shown in Figure 3. The heat produced by this force of attraction can mediate the release of VEGF antibodies in the neighboring tumor cells and blocking the angiogenic pathway. We forecast a problem in locating cancer stem cell on the superficial part of tumour, therefore, the use of magnet can help the nanoparticle to invade to the interior parts of tumour and locate the cancer stem cell. After locating the cancer stem cell only, the nanoparticle will unfold and release the VEGF monoclonal antibody to block endothelial cells near cancer stem cell. This strategy will be specific as normal angiogenesis is not blocked and probability of occurrence of cancer stem cell marker on normal cell is less. Even if the cancer stem cell marker and normal cell marker overlaps, then the use of magnet to tumour site tracks the nanoparticle only to tumour site and not to the normal cells/tissues. Therefore using these multiple ways, we can make the nanoparticle targeting more specific.

## Conclusion

Angiogenesis plays a critical role in the development of cancer, its progression and metastasis. The tumor is composed of heterogeneous cells. The tumor is believed to have a stem cell niche which nurtures the cancer stem cells leading to their division and differentiation into cancer cells. These cancer stem cells have found to be rich in HIF-1α which is a transcriptional factor and it is activated in the hypoxic conditions in the tumor site and is needed for it survival and proliferation. This HIF-1α leads to the expression of VEGFR in the cells. VEGF is a key regulator of angiogenesis in the tumors. Tumour growth occurs due to the presence of cancer stem cell combined with angiogenesis in the proximity of cancer stem cells. Thus an effective method of controlling tumour occurrence/reoccurrence could be targeting endothelial cell near cancer stem cells. As we have discussed above, the use of twin nanoparticle for targeting cancer stem cell niche and VEGF for inhibiting angiogenesis in the cancer stem cell niche. This method will lead to the destruction of CSCs and VEGF mediated pathway of angiogenesis. This twin nanoparticle can be made up of magnetite and gold and both can carry specific antibodies for CSC and VEGFR on their surface. Earlier it was noted that iron nanoparticles can accumulate in the body and can cause toxicity in the body. Recently, iron core gold shell nanoparticle has been reported to be less toxic. Also, the nanoparticles will be carrying a CSC specific marker it will bind specifically to the Cancer Stem cells and VEGF monoclonal antibody/inhibitor will bind to endothelial cell present in/near the tumor site. This can be done by the use of magnetic force which can be applied from the outside. This can act as a remote control to accumulate the nanoparticles in the desired place, i.e. the tumor site. The heat produced by the magnetic attraction between the nanoparticle and the external magnetic force can be used for the release of the VEGF monoclonal antibodies into the tumor site. This method can be very specific with very low toxicity in the body. This new technology using twin nanoparticle offers specific targeting of (VEGF inhibitors) drug to tumor stem cell niche, thereby inhibiting tumour growth in the cancer stem cell niche. This new technology can be better than the classical therapy as the earlier inhibits angiogenesis in tumour nonspecifically and not specifically on cancer stem cell niche.

## Abbreviations

Growth Factor; PDGF: Platelet Derived Growth Factor; MAb: Monoclonal antibody; CSC: Cancer Stem Cell; VEGFR: Vascular Endothelial Growth Factor Receptor; CD: Cluster of Differentiation; TK: Tyrosine Kinase.

## Competing interests

The authors declare that they have no competing interests.

## Authors' contributions

RKA contributed conceptual information. RKA, AS and PK have reviewed the literatures and drafted the manuscript. All authors have read and approved the final manuscript.
